# Posterior acetabular wall morphology is an independent risk factor that affects the occurrence of acetabular wall fracture in patients with traumatic, posterior hip dislocation

**DOI:** 10.1007/s00068-022-02072-0

**Published:** 2022-10-04

**Authors:** Tilman Graulich, Pascal Gräff, Tarek Omar Pacha, Marcus Örgel, Christian Macke, Mohamed Omar, Christian Krettek, Emmanouil Liodakis

**Affiliations:** Trauma Department, Hannover Medical School, Carl-Neuberg-Str. 1, 30625 Hannover, Germany

**Keywords:** Posterior acetabular wall, Traumatic hip dislocation, Fracture, Pipkin fracture

## Abstract

**Purpose:**

Smaller posterior acetabular walls have been shown to independently influence the risk for bipolar hip dislocation. We asked whether differences would also be observed in patients with traumatic posterior hip dislocation with and without posterior wall fractures.

**Methods:**

Between 2012 and 2020 we observed 67 traumatic posterior hip dislocations. Of these, 43 traumatic posterior hip dislocations in 41 patients met the inclusion criteria. Eighteen dislocations were excluded with an acetabular fracture other than posterior wall fracture and six dislocations had insufficient computed tomography (CT) data. The mean age was 41 ± 11 years, 32 males and nine females. We observed 26 traumatic hip dislocations with posterior wall fractures and 17 without. All patients underwent polytrauma CT scans and postoperative/postinterventional pelvic CT scans. On axial CT-scans, posterior wall determining angles were measured.

**Results:**

Patients with posterior wall fractures were not significantly older than patients without posterior wall fractures (42 ± 12 vs. 38 ± 10 years; *p* = 0.17). Patients without posterior wall fractures had significantly smaller posterior acetabular sector angles (84° ± 10°) than did patients with posterior wall fractures (105° ± 12°) (*p* < 0.01; OR 1.178). Likewise, the posterior wall angle was significantly smaller in patients without posterior wall fracture (62° ± 9°) than in those with posterior wall fractures (71° ± 8°) (*p* < 0.01; OR 1.141).

**Conclusion:**

Both posterior acetabular sector angle and posterior wall angle are independent factors determining the posterior wall fracture morphology in patients with traumatic posterior hip dislocation. Age and the observed trauma mechanism did not differentiate between traumatic posterior hip dislocations with and without posterior wall fractures.

## Introduction

Traumatic posterior hip dislocations are most commonly seen as dashboard injuries in car accidents and can occur either with or without fracture of the femoral head and either with or without fracture of the posterior acetabular wall [1, 2]. Concomitant femoral head fractures were observed in 5–15% of all posterior hip dislocations, and posterior wall fractures occurred in about 75% of all posterior hip dislocations [2, 3]. Traumatic posterior hip dislocations have been classified by Thompson and Epstein into five types, beginning with simple dislocation without fracture towards higher degree of fracture with posterior wall fragment, impression of the quadrilateral surface, and femoral head fracture [3, 4]. Posterior wall fractures of the acetabulum are among the most common acetabular fractures [4–6]. Judet and Letuernel stated that the mechanism of posterior wall fracture was direct axial force transmission from the flexed femoral head towards the acetabulum [6]; the flexion degree determined whether the force was transmitted superiorly or inferiorly [6]. Finally, they described two types of posterior wall fracture, with or without posterior comminution of the wall fragment. Comminution is associated with poor outcome [6, 7]. As independent factors determining the degree of fracture and comminution the “height, width, and location” are “functions of the direction and magnitude of the force transmitted to the acetabulum” [6]. Femoral head fractures themselves have been classified by Pipkin into types I–IV [1, 2]. Their inherent nature describes a pathological situation with a traumatic hip dislocation and accompanying different types of femoral head, avulsion-like fractures or femoral neck fractures or fracture of the posterior acetabular wall. Types I–III are femoral head fractures without posterior wall involvement, and type IV fractures are femoral head fractures with posterior wall involvement.

**Table 1:** Trauma mechanism and dislocation type

Mechanism	Traumatic posterior hip dislocation without posterior wall fracture (*n* = 17)	Traumatic posterior hip dislocation with posterior wall fracture (*n* = 26)
Dashboard injury (*n*/%)	12/70	17/65
Motorcycle (*n*/%)	2/12	6/23
Other (*n*/%)	3/18	3/12

Recently, we showed that posterior wall morphology differs by age, gender, and the degree of osteoarthritis [8]. The various posterior wall types can be distinguished by measurement of posterior wall angles [8, 9]. The method was adapted from Valera et al., who analyzed the acetabular shape and described the anterior and posterior acetabular coverage in healthy patients younger than 55 years without higher degrees of osteoarthritis [9]. Likewise, reduced posterior acetabular sector angle (PASA) and reduced posterior wall angle (PWA) were thought to be among the major patient-related specific factors influencing the risk for bipolar hemiarthroplasty dislocation [10].

We assume that the differences in bipolar hemiarthroplasty dislocation and traumatic posterior hip dislocations are based on the same anatomical differences of posterior wall morphology with reduced PASA and PWA. Both PASA and PWA are indirect parameters determining the posterior femoral head coverage in the axial plane. Likewise, in the frontal plane, the center edge (CE-angle) angle described by Widberg et al. can be seen as an indirect parameter characterizing the femoral head coverage [11]. A small CE-angle in the frontal plane increases the peak stress in dysplastic hips up to 7.1 kPa/N compared to healthy hips (3.5 kPa/N), and most importantly, it shifts the peak force from central to a more lateral position [11].

As the joint pressure equals the transmitted force divided by the surface area, a smaller posterior wall surface area is expected to result in a higher joint pressure during traumatic posterior hip dislocation [12]. Therefore, we believe that a smaller posterior wall will increase the acetabular peak force and shift it more laterally. This may lead to dislocation without posterior wall fracture, but resulting in a shear fracture of the femoral head. Second, a larger posterior wall leads to a more central force transmission leading to a posterior wall fracture at the point of force impact in patients with traumatic posterior hip dislocation. The goal of the present study was to answer the following questions: When does the hip dislocate posteriorly with fracture of the posterior acetabular wall, and when does the hip dislocate without fracture of the posterior acetabular wall?

## Patients and methods

**Fig. 1:**
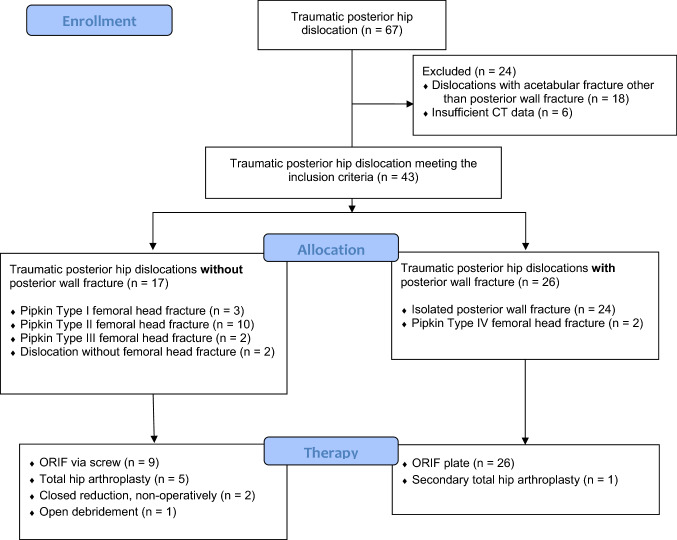
Flow chart enrolment

Between 2012 and 2020, we observed 67 traumatic hip dislocations. Of these, we recorded 43 traumatically dislocated hips in 41 patients. Exclusion criteria were a higher degree of acetabular fracture such as involvement of the posterior column or impression of the quadrilateral surface and missing or insufficient CT data. Overall, 24 posterior hip dislocations were excluded. Of these, 18 traumatic posterior hip dislocations were excluded due to a higher degree of acetabular fracture and six traumatic posterior hip dislocations were excluded due to missing or insufficient CT data (Fig. 1). The mean age was 41 ± years, 32 males and nine females. We observed 26 traumatic hip dislocations with posterior wall fractures and 17 traumatic hip dislocations without posterior wall fractures. All patients underwent polytrauma CT scans and postoperative/postinterventional pelvic CT scans. Fracture morphology was classified according to Pipkin classification, and trauma mechanism was monitored from medical records. Trauma mechanism and operative or non-operative treatment strategies were monitored from medical records.

**Table 2:** Trauma mechanism and acetabular angles

	Dashboard injury (*n* = 29)	Motorcycle injury (*n* = 8)	Other (*n* = 6)	*p* value
Coverage (%)	36.6 ± 9.8	43.2 ± 14.9	39.5 ± 3.1	> 0.05
AAA (°)	17.2 ± 7.2	19.2 ± 7.4	19.2 ± 4.4	> 0.05
PASA (°)	94.1 ± 14.6	102 ± 18.7	104.5 ± 11.5	> 0.05
PWA (°)	66.6 ± 10.5	73.7 ± 9.9	68.3 ± 8.0	> 0.05

**Fig. 2:**
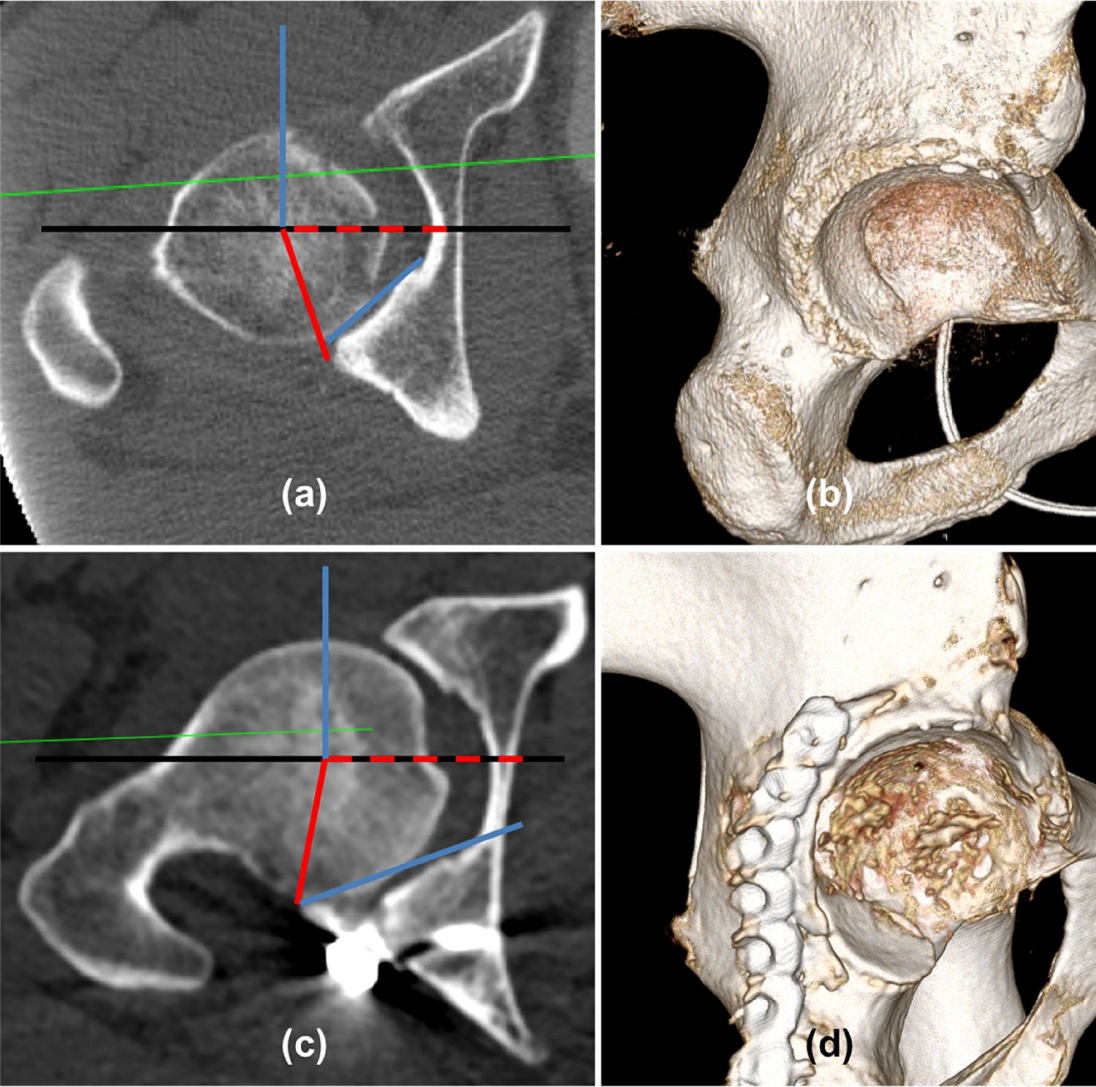
CT-morphological acetabular measurement. **a** and **b** A 35-year-old man with traumatic posterior hip dislocation with femoral head fracture but without posterior wall fracture. **c** and **d** A 34-year-old patient with a traumatic posterior hip dislocation without femoral head fracture but with posterior wall fracture. **a** and **c** Strictly axial images of the right hip. **b** and **d** Strict lateral view on 3D reconstructions of CT scans showing a small and open acetabulum in image **b** and **a** large and closed posterior wall in image **d**. Red dashed line (ICL): line between both femoral head centers, black line: extension of the ICL, blue lines: line perpendicular to the ICL defined as ICL90 and line defined as tangent to the posterior acetabular joint surface. Posterior acetabular sector angle (PASA): angle between both red lines, posterior wall angle (PWA): Angle between both blue lines

To evaluate the acetabular morphology with special respect to the posterior wall, radiological evaluation of axial CT scans included acetabular coverage (%), acetabular anteversion angle (AAA), posterior acetabular sector angle (PASA) and the posterior wall angle (PWA°). The method was adapted from Valera et al. [8, 9] (Fig. 2). A reference line through the center of both femoral heads was drawn (ICL). An orthogonal line through the center of the femoral hip was also drawn (ICL90). A line between the anterior and posterior acetabular lip of the acetabulum (AVL) was drawn to determine the part of the covered femoral head. This part was divided by the total diameter of the femoral head. Data were expressed as % of the total. AAA° was determined by the angle between the ICL90 and AVL. The PASA° was determined by measuring the angle between the ICL and a line from the femoral head center to the lateral edge of the posterior wall [2]. The angle between the ICL90 and the tangent to the posterior articular surface area determined the PWA.

### Statistical analysis

Data were tested for normal distribution using the Shapiro–Wilk test. For comparative statistics, in case of normal distribution, the *t* test was used. If data were not normally distributed, the Mann–Whitney *U* test was used. Data were expressed as median with 25% and 75% quartiles, or if normally distributed, as mean ± standard deviation. Pearson correlation testing was performed for correlation testing of interrater correlation and correlation testing of the measured acetabular angles. A receiver operating characteristic (ROC) analysis was conducted for sensitivity and specificity analysis of the angles.

### Institutional review board approval

Institutional Review Board approval was not required because the investigator conducting this research obtained: (1) no data through interventional interaction; and (2) no identifiable private information.

## Results

Of the 43 traumatic dislocated hips in 41 patients, we observed 26 traumatic hip dislocations with posterior wall fractures and 17 traumatic hip dislocations without posterior wall fractures. Patients with posterior wall fractures were not significantly older than patients without posterior wall fractures (42 ± 12 years vs. 38 ± 10 years, *p* = 0.272).

### Trauma mechanism

Overall, we observed 29 dashboard injuries after car accidents. No differences in the incidence of trauma mechanism between traumatic posterior hip dislocations with or without posterior wall fractures were observed (17/65 vs. 12/70%). Eight patients suffered motorcycle accidents. There were no differences in terms of incidence of trauma mechanism between traumatic posterior hip dislocations with or without posterior wall fractures (6/23 vs. 2/12%). Six patients were grouped as “other mechanism” with one patient hit by a gun shot and five patients who fell from a height larger than three meter (Table 1). There were no differences in terms of incidence of trauma mechanism between traumatic posterior hip dislocations with or without posterior wall fractures. Furthermore, acetabular angles did not differ between patients with different trauma mechanism (Table 2).

**Table 3:** Radiological measurements and dislocation type

	Total (*n* = 43)	Traumatic posterior hip dislocation without posterior wall fracture (*n* =17)	Traumatic posterior hip dislocation with posterior wall fracture (*n* = 26)	*p* value
Coverage (%)	38 ± 10	34 ± 10	41 ± 10	**0.03**
AAA (°)	38 ± 10	15 ± 7	19 ± 6	0.18
PASA (°)	97 ± 15	84 ± 10	19 ± 6	**< 0.01**
PWA (°)	68 ± 10	62 ± 9	19 ± 6	**< 0.01**

Bold values are considered statistically significant with *p*<0.05

### Fracture classification and treatment

In the 17 traumatic posterior hip dislocations without posterior wall fractures, we observed three pipkin type I fractures, 10 Pipkin type II fractures, and two pipkin type III fractures. Two patients suffered dislocations without fractures. Nine dislocated hips underwent open reduction and internal fixation (ORIF) via screws, nine dislocated hips underwent total hip arthroplasty (THA), two dislocated hips underwent closed reposition, and one dislocated hip underwent open debridement. In the 26 traumatic posterior hip dislocations with posterior wall fractures, we observed 24 isolated posterior wall fractures and two Pipkin type IV fractures. All 26 traumatic posterior hip dislocations underwent ORIF via posterior plating of the acetabular wall. One patient had to be converted to THA secondarily (Fig. 1).

### Radiological measurements

Interestingly, patients without posterior wall fractures showed significantly smaller PASA than patients with posterior wall fractures (84° ± 10° vs. 105° ± 12°; *p* < 0.01; OR 1.137). Likewise, the PWA was significantly smaller in patients without posterior wall fractures than in patients with posterior wall fracture (62° ± 9° vs. 72° ± 8°; *p* < 0.01; OR 1.112). The femoral head coverage was significantly smaller in patients without posterior wall fractures than in patients with posterior wall fractures (34° ± 10° vs 41° ± 10°; *p* < 0.03) However, the AAA showed no significant differences between both groups (16° ± 5° vs. 19° ± 7°; *p* > 0.05) (Table 3).

ROC curve analysis showed that a PASA angle of 89° [area under the curve (AUC) 0.906] had a good test result for traumatic hip dislocations with posterior wall fractures with a sensitivity of 92.3% and a specificity of 82.4%. PWA angle of 61.5° (AUC 0.809) showed a fair test result for traumatic hip dislocations with posterior wall fractures with a sensitivity of 88.5% and a specificity of 47.1%.

## Discussion

We observed two major findings: a smaller posterior wall is associated with a higher risk of traumatic posterior hip dislocation without posterior wall fracture, but a high number of shear fractures of the femoral head and vice versa larger posterior walls are associated with higher risk of traumatic posterior hip dislocations accompanied by posterior wall fractures. Both PASA and PWA are adequate parameters to distinguish between patients who will have a higher risk of suffering either a traumatic poster hip dislocation with posterior wall or femoral head fracture. Second, age did not determine fracture morphology. Furthermore, the fracture mechanism did not differ significantly between traumatic posterior hip dislocations with and without posterior wall fractures.

Our hypothesis was based on the assumption that, in the axial plane, similar biomechanical properties can be observed as in the frontal plane with respect to axial force transmission via the femur towards the acetabulum. Furthermore, we assumed that both PASA and PWA would describe the posterior wall morphology in the axial plane, as the CE-angle does in the frontal plane. In other words, we assumed that a decrease in CE-angle would be associated with (1) greater peak stress than in patients with normal CE angles and (2) with shifts of the peak force from central to a more lateral position, analogously small PASA would increase the peak force and shift the peak force from central to lateral in the axial plane [11].

As shown by Vukasinovic et al. who compared hip forces before and after triple pelvic osteotomy in the frontal plane, comparing biomechanical forces in case of preoperative small- and postoperative larger CE-angle, significant changes in hip forces were observed. Most interestingly by enlargement of CE-angle via triple pelvic osteotomy (1) peak contact hip stress normalized to body weight was decreased by 55.9% and (2) the vector of the stress pole shifted medially, by 63%. We believe that a larger more centralized peak force will more likely result in a fracture of the posterior acetabular wall with or without shear fracture of the femoral head in patients with a larger posterior wall than in patients with a smaller posterior wall. Likewise, a more open posterior acetabular wall increases the shear stress to the femoral head letting it slip out of the cup by means of dislocation either with or without fracture of the femoral head but most probably resulting in labral tears but no posterior wall fracture [13].

We are aware of the missing biomechanical examination in our study, which is certainly one major limitation of our study. Nevertheless, despite the fact that a causative association of fracture morphology and posterior wall morphology in patients with traumatic posterior hip dislocation could not be determined in our study, we can state that a significant correlation of posterior wall morphology and fracture morphology can be made with a sensitivity of up to 88.9%. This is in line with our previous observations in patients with posterior bipolar hip arthroplasty dislocations [10].

As shown by Judet and Letournel, the fracture morphology of the posterior wall depends on the location of the femoral head with respect to the acetabulum, and by this, the concomitant and axial force transmitted to the acetabulum. Whereas a more flexed and adducted femur will lead to an inferior and more outer fracture, a more extended and abducted femur will lead to a more superior and more central posterior wall fracture [6]. Due to the retrospective nature of our study and the missing biomechanical evaluations of the fracture mechanism, the impact of possible differences in force transmission towards the hip joint leading to different fracture morphologies cannot be clearly identified and may be another limitation of this study. Furthermore, the impact of proximal femoral morphology on the risk of hip dislocation was not observed in this study and is another limitation. As Steppacher et al. have stated femoroacetabular impingement predisposes to posterior hip dislocation [14]. Most interestingly a cam-type impingement might act like a fulcrum. Unfortunately, Steppachers et al. and likewise our data, both lake information on femoral torsion due to missing whole leg CT-scans. This would most likely help to analyze the femoroacetabular anatomy, influencing the morphological differences in patients with posterior hip dislocation. Although femoral morphological differences have not been included in this study, we believe that the differences in acetabular morphology are an individual factor influencing posterior hip dislocations with- and without posterior wall fracture. We could show that besides the in the literature known reduction of the acetabular anteversion, which was not statistically significant different between our study groups, the size of the posterior wall determines the type of posterior wall dislocation [14].

Normal hip contraction forces have been described previously [15–17]. In healthy hips, the normal hip power in a single leg stand is about 1500 N, whereas in dysplastic hips, this power rises up to 2295 N [18]. In dysplastic hips, the center edge angle is an important parameter to determine the size of the weight-bearing surface and is associated with the development of osteoarthritis [11, 19]. This is an indirect parameter to determine the hip stress. As in the frontal plane, the CE-angle defines the cranial acetabular coverage; the PASA angle likewise defines the posterior acetabular coverage in the axial plane [20]. A decrease in CE-angle is directly associated with a decrease of cranial acetabular contact area. In this manner, an increase of peak pressure contact force can be observed [20].

Finally questioning the clinical relevance of our results, we believe (I) that in general our data clearly give reason to focus on posterior wall morphology in various pathologic situations at the hip by means of analyzing combined acetabular and femoral version to better understand the pathomorphological basics and (II) to our mind the question arises weather the posterior acetabular wall morphology is an independent factor for posttraumatic osteoarthritis and by this weather an initial early arthroplasty might be an better option in some patients or alternatively a slight correction of the acetabular posterior wall in the intraoperative, early traumatic situation might be an adequate tool. The analysis of labral and cartilage damages seems to be one further parameter which might differ between patients with traumatic hip dislocation either with- or without fracture of the posterior acetabular wall. Therefore, to our mind further studies need to focus on differential labral and cartilage damages in both traumatic situations. Either the focused intraoperative analysis or a preoperative MRI of the injured hip might be adequate tools to determine labral and cartilage damages, address them adequately and to identify options to reduce the rate of posttraumatic osteoarthritis in patients with traumatic hip dislocation either with- or without fracture of the posterior acetabular wall.

## Conclusion

A smaller posterior wall is associated with a higher risk of traumatic posterior hip dislocation without posterior wall fracture and a high number of shear fractures of the femoral head. A larger posterior wall is associated with a higher risk of traumatic posterior hip dislocations with posterior wall fractures. Both PASA and PWA are appropriate parameters to distinguish between patients who will have a higher risk of suffering either traumatic posterior hip dislocation with posterior wall fractures or with femoral head fractures.

## References

[1] Epstein HC, Wiss DA, Cozen L (1985). Posterior fracture dislocation of the hip with fractures of the femoral head. Clin Orthop Relat Res.

[2] Romeo NM, Firoozabadi R (2018). Classifications in brief: the pipkin classification of femoral head fractures. Clin Orthop Relat Res.

[3] Epstein (1973). Traumatic dislocation of the hip. Clin Orthop Relat Res.

[4] Smith GR, Loop JW (1976). Radiologic classification of posterior dislocations of the hip: refinements and pitfalls. Radiology.

[5] Firoozabadi R, Chen EY, Elhaddad M, Tornetta P (2020). Isolated buttress plating of posterior wall acetabular fractures: is it sufficient?. Arch Bone Jt Surg.

[6] Judet R, Judet J, Letournel E (1964). Fractures of the acetabulum: classification and surgical approaches for open reduction. Preliminary report. J Bone Jt Surg Am.

[7] Olson SA, Bay BK, Chapman MW, Sharkley NA (1995). Biomechanical consequences of fracture and repair of the posterior wall of the acetabulum. J Bone Jt Surg Ser A.

[8] Graulich T, Graeff P, Nicolaides S (2020). Acetabular posterior wall morphology. A CT-based method to distinguish two acetabular posterior wall types. J Orthop.

[9] Valera M, Ibáñez N, Sancho R (2018). Acetabular overcoverage in the horizontal plane: an underdiagnosed trigger of early hip arthritis. A CT scan study in young adults. Arch Orthop Trauma Surg.

[10] Graulich T, Graeff P, Nicolaides S (2021). Risk factors for dislocation after bipolar hemiarthroplasty: a retrospective case–control study of patients with CT data. Eur J Orthop Surg Traumatol.

[11] Mavčič B, Pompe B, Antolič V (2002). Mathematical estimation of stress distribution in normal and dysplastic human hips. J Orthop Res.

[12] Polkowski GG, Clohisy JC (2010). Hip biomechanics. Sports Med Arthrosc.

[13] Vukasinovic Z, Spasovski D, Kralj-Iglic V (2013). Impact of triple pelvic osteotomy on contact stress pressure distribution in the hip joint. Int Orthop.

[14] Steppacher SD, Albers CE, Siebenrock KA (2013). Femoroacetabular impingement predisposes to traumatic posterior hip dislocation. Clin Orthop Relat Res.

[15] Bergmann G, Bergmann G, Deuretzabacher G (2001). Hip forces and gait patterns from routine activities. J Biomech.

[16] Heller MO, Bergmann G, Deuretzbacher G (2001). Musculo-skeletal loading conditions at the hip during walking and stair climbing. J Biomech.

[17] Paul JP (1976). Force actions transmitted by joints in the human body. Proc R Soc Lon B Biol Sci.

[18] Carls J, Wirth CJ, Börner C, Pape A (2002). Änderung der biomechanik dysplastischer hüftgelenke durch implantation einer hüfttotalendoprothese. Z Orthop Ihre Grenzgeb.

[19] Cooperman D (2013). What is the evidence to support acetabular dysplasia as a cause of osteoarthritis?. J Pediatr Orthop.

[20] Genda E, Iwasaki N, Li G (2001). Normal hip joint contact pressure distribution in single-leg standing-effect of gender and anatomic parameters. J Biomech.

